# Squamous cell carcinoma of a colon transplant 29 years after restorative esophagoplasty for caustic ingestion

**DOI:** 10.1016/j.ijscr.2025.111065

**Published:** 2025-02-17

**Authors:** Iliass Maouni, Youness Bakali, Ahmed Jahid, Mohamed El Absi, E.H. El Alami, Sarah Benammi

**Affiliations:** a University Hospital Ibn Sina, Rabat, Morocco; b Mohamed V University of Rabat; c Mohamed VI University of Sciences and Health; d International University Hospital Mohamed VI, Bouskoura, Morocco

**Keywords:** Esophagoplasty, Colon transplant, Degeneration, Squamous cell carcinoma, Long-term follow-up

## Abstract

**Introduction:**

We aim to report a case of squamous cell carcinoma 29 years following restorative colonic transplant in esophagoplasty for caustic ingestion and to emphasize the importance of long-term follow-up.

**Presentation of case:**

We report a case of a 59-yo patient with malignant degeneration of colon transplant squamous cell 29 years following restorative esophagoplasty for caustic ingestion. He reported symptoms of progressively worsening dysphagia, odynophagia, and left cervical mass with fistula. The assessment revealed a squamous cell carcinoma of the colon graft without a distant lesion.

**Discussion:**

Esophagoplasty by colonic transplant is a widely used surgical technique for the treatment of benign or malignant lesions of the esophagus. The degeneration of colonic transplant is exceptional, most reportedly into adenocarcinoma. Squamous cell carcinoma is very rare and scarcely reported. Preoperative colonic segment assessment is mandatory. Follow-up includes essentially endoscopic evaluation, which allows both visual inspection and histologic evaluation of the transplant.

**Conclusion:**

The risk of carcinogenesis in colonic transplants after esophagectomy is exceptional. The cases published in the literature are scarce. Although rare, this risk justifies a long-term follow-up of these patients by performing an annual upper GI endoscopy.

## Highlights

• Esophagoplasty via colonic transplant treats esophageal lesions; pre-op assessement is vital. Long term follow-up is mandatory.

## 1 Introduction

The use of large bowel to replace the esophagus was first introduced in 1911 [1,2]. The colon became afterward the most used organ for esophageal reconstruction in adults for benign or malignant esophageal lesions [3]. Colonic transposition underwent several modifications of the technique using the different segments as an interposition graft [4].

Late complication of esophageal substitutes is rarely described and mainly include morphologic and functional complication, especially when using colonic transplant in esophagoplasty [5]. The relatively few late sequelae after substernal colonic esophageal transplant may be successfully managed surgically [5]. Overall colon substitute is an effective option allowing gastrointestinal continuity with reduced mortality and good functional outcome and health perception in the long-term [6]. Risk of malignant transformation of the colonic transplant after esophagectomy is exceptional [7]. The cases published in the literature are very few and mainly were described adenocarcinoma cases [7].

We present the case of a 59-year-old patient who developed malignant degeneration into squamous cell carcinoma in a colonic graft, 29 years after undergoing restorative esophagoplasty for caustic ingestion. We aim to emphasize the importance of long-term follow-up.

## 2 Presentation of case

This was a 59-year-old patient with a history of resolutive pulmonary tuberculosis in 1995 and 2 small bowel obstruction due to adhesions history. At the age of 30 years old the patient was admitted for management of unintentional caustic ingestion, undergoing esogastrectomy with left colonic transplant after preoperative assessment. The patient was not consistent with medical follow-up after his esogastrectomy.

**Fig. 1:**
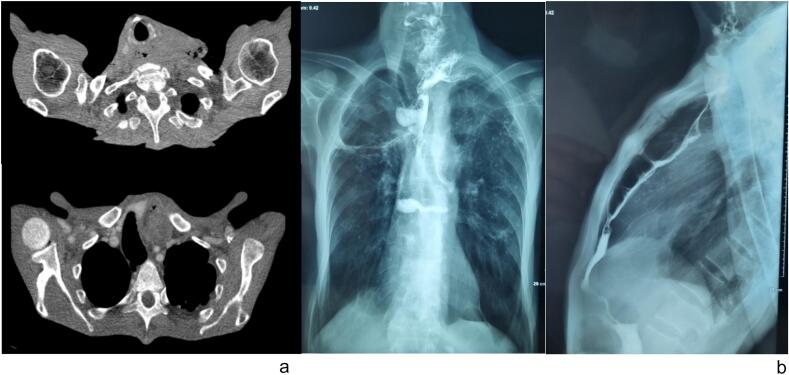
(a) CT view showing distension of the oesophageal plasty at the left cervical level with bubbles of extra-digestive areas without extravasation of contrast. (b) Barium swallow showing heterogeneous aspect of the colonic esophageal plasty with 2 blind fistulas of the left cervico-thoracic junction

The patient reported history of dysphagia associated with worsening odynophagia for the last year, with fistulized left cervical mass and weight loss. Abdominal cervical and thoracic CT scan with contrast objectified a parietal thickening of the cervical segment of the colonic esophagoplasty suspected of malignancy with suspected supraclavicular adenopathy (Fig. 1a). Barium swallow was performed reporting a heterogeneous appearance of the colonic esophagoplasty with 2 fistulas of the left cervico-thoracic junction (Fig. 1b). Upper digestive endoscopy showed the presence at the level of the coloesophageal anastomosis of an ulcerative and vegetative tumoral process 5 cm in long axis without stenosis. Biopsy showed a mature and keratinizing invasive squamous cell carcinoma. Biopsy of the cervical mass did not conclude having malignant cells. Staging showed no signs of regional spread or metastatic disease.

Treatment protocol was established after multidisciplinary tumor board assessment. The patient was proposed for jejunostomy and neoadjuvant radiotherapy, followed by re-staging and consideration of surgical removal of the tumor. The patient refused surgical management including jejunostomy and was lost at follow-up. Consent was collected upon announcement of diagnosis. Written informed consent was obtained from the patient for publication of this case report and accompanying images. A copy of the written consent is available for review by the Editor-in-Chief of this journal on request. Our case report has been reported in line with the SCARE criteria [8].

## 3 Discussion

Bowel interposition after esophageal resection is a technique that has been widely used for more than a century. Early complications of this procedure are frequent, namely anastomotic fistulas, stenosis and necrosis of the colonic graft [9]. Late complications, on the other hand are rare. Cases of gastroduodenal colitis, stricture of the colonic graft, gastro-colic reflux or colo-bronchial and colo-pericardial fistulas have been described previously in the literature [10,11].

Malignant degeneration of the colonic graft is an exceptional complication. A review of the literature reports a very few published cases (less than 10) [7]. In all cases, the histological type reported was adenocarcinoma [7,12]. Dysphagia was the most reported symptom, less frequently reflux symptoms and respiratory infection [7]. Diagnosis and staging were defined by gastrointestinal endoscopy allowing biopsy confirmation, and CT scan with eventually PET scan [7]. Resection of the interposed colon was the procedure of choice for cure [7].

We report a rare case of squamous cell carcinoma degeneration of the colonic transplant. This histological type could be explained by several theories. Exposure to undigested food and reflux of intestinal juices can induce squamous cell carcinoma metaplasia at the level of the upper aerodigestive tract, in addition to the hypopharynx, which would have invaded the colonic transplant; or squamous metaplasia in the colonic graft [13,14]. The environmental changes that occur to an interposed colon, in contrast to its natural milieu, may help promote dysplastic change and augment precancerous conditions [7]. However the sequence metaplasia-dysplasia-squamous cell carcinoma has yet to be determined in our case.

In fact considering the malignant transformations of the colonic interposition grafts are mostly adenocarcinomas, we believe that the squamous cell carcinoma was so most likely an upper esophageal degeneration with downward growth at anastomotic level. Unfortunately, the hypothesis cannot be sustained due to the refusal of surgical care by the patient and lack of more detailed histological study.

Although this phenomenon of carcinogenesis is still rare, any late dysphagia in a patient with colonic plasty after esophagectomy should be thoroughly investigated. Colonoscopy assessment of the transplant segment is therefore set to be vital in order to rule out contraindications for use in osephagoplasty [4]. Moreover, long-term follow-up is mandatory. The barium transit may be difficult to interpret because of the change in the patient's anatomy [15,16]. Follow-up is mainly upper GI endoscopy, which allows to evaluate the macroscopic aspect of the observed lesions and to perform biopsies. Once the diagnosis of malignancy is confirmed on histology, the thoraco-abdominal pelvic CT scan performed as part of the extension workup remains the examination of choice to explore the invasion of neighboring organs and to search for pulmonary and hepatic metastases. It has been suggested that colon transplants follow-up should be annual and follow the age adjusted incidence rate of colon cancer [7,17]. Although our clinical case was not completed due to the refusal of our patient, we opted after multidisciplinary tumor board assessment for jejunostomy and neoadjuvant radiotherapy, followed by re-staging and surgical removal of the tumor despite squamous carcinoma histology considering that the graft was a colon segment, therefore removal of the graft and the anastomosis segment was preferable. However, surgery at this level is difficult with high morbidity, and exclusive chemoradiation therapy after creation of feeding stoma can be considered.

## 4 Conclusion

Carcinogenesis of the colonic transplant after esophagectomy is exceptional rare, therefore more prevalent studies are required. The cases published in the literature are very few. Although it is rare with very scarce data available in literature, this risk justifies a long-term follow-up of these patients by performing an annual upper GI endoscopy.

## Consent

Written informed consent was obtained from the patient to publish this case report and accompanying images. On request, a copy of the written consent is available for review by the Editor-in-Chief of this journal.

## Ethical approval

This case report is exempt from ethical approval in our institute.

## Sources of funding

This was a case report with no funding required or obtained.

## Financial disclosure

None to declare.

## Declaration of competing interest

We have no conflict of interest to declare.
